# Diagnostic Delay for Head and Neck Cancer in South India: A Mixed-Methods Study

**DOI:** 10.31557/APJCP.2020.21.6.1673

**Published:** 2020-06

**Authors:** Sivaraman Ganesan, Sivanesan Sivagnanaganesan, Mahalakshmy Thulasingam, Gunaseelan Karunanithi, Kalaiarasi R, Surya Ravichandran, Sunil Kumar Saxena, Karthikeyan Ramasamy

**Affiliations:** 1 *Department of ENT, Jawaharlal Institute of Postgraduate Medical Education and Research, Puducherry, India. *; 2 *Department of Preventive and Social Medicine, Jawaharlal Institute of Postgraduate Medical Education and Research, Puducherry, India. *; 3 *Department of Radiation Oncology, Jawaharlal Institute of Postgraduate Medical Education and Research, Puducherry, India. *

**Keywords:** Head and neck cancer, primary delay, secondary delay, symptom appraisal, health-seeking behavior

## Abstract

**Background:**

Early diagnosis is an important aspect of quality of cancer care.Analysis of the diagnostic delays and the reasons for delay helps to plan strategies to improve cancer care.

**Objectives:**

To determine the primary, secondary, and total diagnostic delay of patients diagnosed with head and neck cancer and to explore the reasons for the delay from the patient perspective.

**Methods:**

Explanatory mixed method design was used. Two hundred persons with a confirmed diagnosis of head and neck cancer attending the ENT (ear, nose, throat) cancer clinic in a teaching hospital before the initiation of treatment were included in the study. The median delay and the association of the delay with the various factors were analyzed. Sixteen one-to-one interviews of patients were done to identify the reasons for the delays from the patient perspective.

**Results:**

Median primary, secondary, and total diagnostic delays were 30 days, 30 days, and 73 days, respectively. Statistically, primary delay was found significantly longer among ever users of smokeless tobacco and significantly longer secondary delay was found among those with age less than 60 years. The reasons for the delay were grouped in the categories (i) Symptom appraisal delay due to low perceived seriousness and (ii) health-seeking behavior delay.

**Conclusions:**

The diagnostic delay was considerable. Measures to enhance symptom appraisal by improving health literacy, opportunistic screening, and strengthening the referral system would decrease diagnostic delay.

## Introduction

Head and neck cancer includes malignant neoplasm of lip/oral cavity, salivary gland, nasopharynx, oropharynx, hypopharynx, and larynx. In 2018, the global age-standardized incidence rate (ASR) for head and neck cancer was 10.1 per 1,00,000 population. In India, the ASR for head and neck cancer is higher, 11.5 per 1,00,000 population (International Agency for Research on Cancer, 2018). In India, 60-90% of head and neck cancer present at the late stage of the disease.(Kulkarni, 2013; Singh et al., 2015).

Delay in cancer diagnosis and treatment adversely impact survival, recurrence rate, cost of treatment and quality of life of patients. To achieve Sustainable Developmental Goal (SDG) target to reduce by one-third premature mortality from non-communicable diseases by 2030, the World Health Organization (WHO) recommends ‘Comprehensive Cancer Control.’ One of the core components of ‘Comprehensive Cancer Control’ is early diagnosis. Early diagnosis indicates early identification of cancer among persons with symptoms consistent with cancer. Multiple models have been proposed to understand the delay in seeking medical care; the simplified framework put forth by WHO is linked with actionable recommendations (Anderson et al., 1995; Safer et al., 1979; WHO, 2017). WHO identifies three essential steps for cancer early diagnosis: step 1: awareness and accessing care, step 2: clinical evaluation, diagnosis and staging, Step 3: access to treatment.The first step ‘awareness and accessing care’ includes (i) symptom appraisal and (ii) health-seeking behavior.

“*Symptom appraisal indicates the period from detecting a bodily change to perceiving a reason to discuss the symptoms with a health-care practitioner; and (ii) health-seeking behavior indicates the period from perceiving a need to discuss the symptoms with a health-care practitioner to reaching the health facility for an assessment.*” The second step, ‘*clinical evaluation, diagnosis and staging*,’ includes accurate clinical diagnosis, diagnostic testing and staging, and referral for treatment (WHO, 2017). These durations are primarily affected by social and cultural contexts (Andersen et al., 2009); hence, WHO recommends situational analysis of obstacles for early diagnosis before planning or scaling up early diagnosis (WHO, 2017).

This study was done to determine the primary, secondary, and total diagnostic delay of patients diagnosed with head and neckcancer and to explore the reasons for the delay from the patient perspective. 

## Materials and Methods

Study design: This is a sequential explanatory mixed-method study(QUAN-qual: QUANTITATIVE – hospital-based descriptive study, Qualitative – descriptive). The qualitative part was used to understand the reasons for patient-related delays. 

*Study setting*

The study was done in the Jawaharlal Institute of Postgraduate Medical Education and Research (JIPMER), a teaching tertiary care setting in Puducherry, South India, in 2016-17. The hospital has an average outpatient attendance of 7,400/month and 2,044 in-patient beds. The institute also has the Regional Cancer Centre. The hospital mostly caters to people from Puducherry and Tamil Nadu. Most of the services are free of cost. The majority of patients belong to low socioeconomic status and have low literacy. Puducherry and Tamil Nadu have good health indicators and better primary health care infrastructure as compared to most other states in India.(National Institution of Transforming India; Ministry of Health and Family Welfare; The World Bank, 2019)

Study population:The study population was Head and Neck cancer patients of age more than 18 years attending the ENT cancer clinic. Patients with a confirmed cancer diagnosis participated in the study before the treatment initiation. Patients who were seriously ill and could not speak were excluded from the study. 

*Sample size*

The sample size was calculated using the formula:

$\frac{{Z_{\alpha /2}}^{2\sigma^{2}}}{E^{2}}$

The following values were used:Z_(α/2)_ = 1.96, mean duration of delay in seeking care as 30 days(Dwivedi et al., 2012), SD as 13 and absolute precision as 6. The sample size was calculated as 200. For the qualitative component, the sample size was 16 one-to-one interviews as we reached data saturation with the sample. The 16 patients were selected purposively to include willing and vocal patients with varied time from the onset of symptoms to seeking health care.

*Procedure*

After obtaining written informed consent, the subjects were interviewed using a pre-tested questionnaire which included details on socio-demographic characteristics, date of onset of symptoms, date of consultation with a doctor, date of advice to the patient on referral to JIPMER, date of consultation at JIPMER, date of diagnosis by biopsy. Using this data the following event intervals were calculated: duration between the patients’ first symptom and consultation with a doctor, duration taken at the health care facilities consulted before reaching JIPMER, duration taken by the patient from advice on referral to the first consultation at JIPMER, duration from the first consultation at JIPMER to confirmation of diagnosis. 

Patients who were vocal, willing to spend the needed time for the interview, with a primary delay of more than two weeks, were purposively selected for the one-to-one interview. The interview schedule was prepared and was reviewed by the authors (SG and MT). The interview was done in a room with adequate privacy and was done by a field staff trained in interview techniques. The interview was audio-recorded and transcribed within a week. 

*Operational definition*

Primary diagnostic delay is defined as the time interval between the patient’s first awareness of symptom or sign to the first consultation with a health care provider.Secondary diagnostic delay is defined as the time interval between the patient’s first consultation with a health care provider to the date of the final histological diagnosis.Total diagnostic delay is the sum of the primary and secondary delay (Seoane et al., 2012) 

*Data analysis*

The data was entered in EpiData Version 3.1. Data analysis was done using EpiData Analysis Version 2.2. The various time durations of primary and secondary delay weresummarized as Median and Inter Quartile range as they were non-normally distributed. Stratified analysis was done for the strata based on age, gender, cancer stage at diagnosis, substance abuse, and facility first contacted. The statistical significance in primary and secondary delay between the different strata was tested using the Mann-Whitney test. 

Thematic analysis of the transcribed interviews was done. Statements were the unit of analysis. Both inductive and detective method was used to make the codes. Similar codes were clubbed into categories and related categories into themes. The analysis was done by the third author (MT) trained in qualitative research and was reviewed by the first author. A few statements for each code are presented in the results. 

The study was approved by the Institute Scientific Advisory Committee and the Institute Ethics Committee. 

## Results

*Quantitative*

Two hundred participants were included in the quantitative survey, and their characteristics are described in Table 2. The majority of them were males (70.5%) and were of age < 60 years (64.5%). Around 50% were using either tobacco or alcohol. The oral cavity (60%) was the common site of malignancy. About 85% were in an advanced stage of cancer (stage III/IV). 

Table 1 summarizes the various diagnostic delays of the study participants. The median diagnostic delay was 73 days (IQR: 47-129). The median primary diagnostic delay was 30 days (IQR: 15-60), and the median secondary diagnosticdelay was 30 days (IQR:19-54). Both primary and secondary diagnostic delays contributed similarly to the total diagnostic delay. 

In stratified analysis, it was noted that there was no statistically significant difference in the duration ofdiagnostic delay between different strata divided by gender, tumor site, stage, and type of facility first contacted. This could also be due to small numbers in each of the strata. Age less than 60 years orthe use of smokeless tobacco were significantly associated with a delay in cancer diagnosis (Table 2). In the study center, eligibility for free service was based on self-reported income. Hence, eliciting true family income was challenging and income was not analyzed.

*Qualitative*

Sixteenparticipants were interviewed, of which thirteen were males. said: “There was no pain, so I did not go to the hospital. I developed pain only after six months. Then I came to the hospital.” Participants’ age ranged from 40 to 78 years of age. The time taken to travel from their residence to JIPMER ranged from one hour to 10 hours. The codes, categories and themes of the qualitative data are summarized in Table 3. The codes were clubbed into two categories: (i) Symptom appraisal delay due to low perceived seriousness and (ii) health-seeking behavior delay. 

*Category 1: Symptom appraisal delay due to low perceived seriousness*

Code1.a-No pain:As the swelling was painless,the patients and their family members did not give importance to the swelling or ulcer. A 65-year-old male participant said: “*There was no pain, so I did not go to the hospital. I developed pain only after six months. Then I came to the hospital.*”

Code 1.b-Belief that symptoms are due to heat in the body (pitta as per Ayurveda): A woman employed as salt worker told: “*I thought the swelling is because my body got heated working in the salt field. I took medicines from a medical shop. With medicines, the swelling used to decrease and again grow big...*” 

Code 1.c-Attributed symptoms to addiction:The guilt of addictions, made the participants feel that the symptoms were due to their addiction and did not seek medical care. This perception also existed with the family member. *“I was not able to eat or swallow. I did not know that I have a mass in the throat. My wife thought it is because of my alcohol and smoking habits. She did not take me to a hospital”* (60yr, male). *“I chew betel nut; I thought that the swelling is because of it. I took treatment from a medical shop. I came to the hospital only when I had pain”* (40yr, female).

Code 1.d-Regrets on low health literacy:The patients regretted seeking health care late as they were not aware that the early warning symptoms of cancer. They also lamented that others in the village were not aware of the symptoms of cancer. Participants were ready to seek care in spite of all the hurdles if they were aware of the seriousness of the condition. *“I stay in a village; I was not aware of the nature of this swelling and its pain. If I knew this could cause severe pain, I would have called someone to accompany me to the hospital”* (58yr, female).*“If anybody had told me that the size of the mass will increase gradually and that it would be cancer, I would have gone to the hospital in spite of my poverty. I would have borrowed money from someone to seek medical care”* (57yr, male).

*Category 2: Health seeking behavior delay*

Code 2.a-Non-affordable health care cost: Patients could not afford for the direct medical and non-medical cost. Non-affordability made the patients change the health facility and contributed to the delay in diagnosis and treatment. Women, old aged patients who needed a person to accompany them to the hospital. This was difficult because of their low socio-economic status and the daily-wage nature of the jobs of the family members. 

“*My son is a Farmer. Since we are poor, he has to earn daily wages; he couldn’t bring me to the hospital. If we don’t go towork ,we don’t get food and so they didn’t take care. My wife is innocent and ill, and she couldn’t accompany me to the hospital. My neighbors also didn’t bring to the hospital*”(70 yr, male).

*“They asked Rs 15,000 for testing. I did not have money, so I did not go there again. I went to a government hospital. There they asked me to go to JIPMER” (74 yr, male).*

Code 2.b-Poor Social Support:One of the reasons for the delay in health-seeking was they could not find a person to accompany them to the hospital especially elderly persons. A 65-year-old male commented,“*Nobody was available to bring me to hospital. If somebody got me to the hospital I would have immediately come after I noticed a swelling*”.The language barrier and low social support due to migration also contributed to delayed health-seeking *“My house is in Tamil Nadu.* *I was working in Bangalore. When I went to a hospital there, they didn’t understand me completely. I also didn’t understand what they told me*” (57yr, male).

Code 2.c-Self remedy: Belief in self-medication and apprehension to visit hospital was also the reason for the delay in some participants. A 58-year-old woman commented,“*I didn’t want to go to hospital… I didn’t tell properly about the symptoms to my family. Since I don’t go to a hospitalthey also too took it easy and didn’t bring me to hospital. I’m afraid of hospitals. If I suffer from fever, cough and cold, I take ‘kashayam’ (an herbal drink)*”.Another woman said,“*I have not gone to a hospital at all. Only twice have I visited a hospital in my life. If I am not well, I take nandurasam (soup with crab). I am afraid of injection and operations…*” (48yr). 

Code 2.d-Self medication: Participants took self-medication from the pharmacy for their symptoms. A 69-year-old male said,“*I thought that it is justswelling. I didn’t know it will become like this. I take alcohol and smoke. I used to have a small ulcer and a burning sensation. I used to take medicines from medicals (pharmacy). If I knew it could be cancer, I would have gone to a hospital*”. Another person commented, “*I used to buy the medicines using the prescription slip of the previous consultations. After taking it for 2 hours, I will be pain-free...*” 

“*Telling others about my health… what is going to happen? So, I didn’t tell about the symptoms to anybody.* *Later, when I said to my family, they said to me to go to the hospital instead of accompanying me. Everybody is busy with their jobs. We had issues with money, and my family had to go to work. They didn’t bring me to the hospital. My friend, who comes to the hospital for his sugar (diabetes) treatment, brought me here along with him”* (60 yr, male).

The above statements also show that multiple factors contributed to delay in health-seeking in the patient and patients sort health care when the symptoms could not be tolerated. But they also confirmed that if they were aware that the symptoms or swelling could be cancer, they would have sort care. 

**Table 1:** Diagnostic Delay of Head and Neck Cancer Patients Attending the Cancer Clinic of the Department of ENT of JIPMER, n=200

Delays	Median in days (IQR)
Primary Delay (a)	
a. Duration from the onset of symptom to consultation at a health care facility	30 (15-60)
Secondary Delay (e)	
b. Duration taken at the health care facilities consulted before reaching JIPMER*	4 (1-14)
c. Duration taken by the patient from advice on referral to the first consultation at JIPMER*	10 (2-19)
d. Duration from the first consultation at JIPMER to confirmation of the diagnosis by histopathology	10 (6-14)
e. Duration from the consultation at a health care facility to confirmation of the diagnosis by histopathology (b+c+d)	30 (19-53)
Total Diagnostic Delay (a+e)	73 (47-129)

* for twenty-one participant first facility contacted was JIPMER and for them, the duration ‘b’ and ‘c’ was noted as zero

**Table 2:** Diagnostic Delay and Its Association with the Socio-Semographic and Clinical Characteristics of Head and Neck Cancer Patients Attending the Cancer Clinic of the Department of ENT in a Medical College, n=200

Characteristics of participants	n	(%)	Primary delay, Median in days (IQR)	Secondary delay, Median in days (IQR)	Diagnostic delay, Median in days (IQR)
Total	200	(100)	30 (15-60)	30 (19-54)	73 (47-129)
Gender					
Male	141	(70.5)	30 (15-60)	30 (21-55)	74 (48-129)
Female	59	(29.5)	30 (15-60)	30 (15-47)	72 (45-128)
Age					
< 60 years	129	(64.5)	30 (15-60)	31 (19-63)^b^	75 (45-123)
> 60 years	71	(35.5)	30 (20-90)	30 (18-44)^b^	69 (49-135)
Smoking form of tobacco					
Ever users^a^	91	(45)	30 (15-60)	32 (20-65)	75 (49-124)
Never users	109	(54)	30 (15-72)	30 (18-46)	72 (45-132)
Alcohol Use					
Ever users^a^	98	(49)	30 (15-60)	30 (19-58)	72 (48-135)
Never users	102	(51)	30 (15-60)	30 (20-48)	74 (46-123)
Smokeless tobacco use					
Ever user^a^	96	(48)	30 (20-120)^c^	28 (14-41)	78 (45-161)
Never users	104	(52)	25 (15-49)^c^	34 (22-67)	70 (49-112)
Site of lesion					
Oral cavity	119	(60)	30 (20-65)	30 (15-47)	70 (47-137)
Oro pharyngeal cancer	23	(12)	30 (10-60)	25 (16-54)	75 (45-121)
Laryngeal cancer	58	(29)	23 (15-60)	32 (22-64)	74 (46-120)
Composite stage of cancer					
I	7	(3)	45 (30-65)	21 (8-29)	85 (51-116)
II	24	(12)	20 (12-60)	31 (23-41)	60 (41-116)
III	49	(25)	21 (15-60)	31 (22-58)	74 (47-116)
IV	120	(60	30 (16-63)	30 (17-56)	75 (47-143)
Facility first contacted					
Private clinic	101	(51)	30 (15 - 60)	30 (20-54)	74 (46-122)
Govt. tertiary care hospital	58	(29)	30 (16 - 60)	37 (24-62)	72 (52-129)
JIPMER	21	(10)	60 (28-145)	12 (9 - 27)	89 (35-166)
Private hospital	10	(5)	25 (15 - 60)	30 (24-34)	54 (44 - 91)
Private medical college	10	(5)	30 (8 -120)	40 (13-63)	88 (42-185)

^a^, ever users include past and current users of the addictive substance mentioned; ^b^, significant with p value=0.03 using Mann-Whitney test; ^c^, significant at *P*-value <0.001 using Mann-Whitney test; IQR, Inter Quartile Range

## Discussion

This study showed that the median diagnostic delay for head and neck cancer among the study participants was 73 days. Primary and secondary delays were similar with a median delay of 30 days. Those who were ever users of smokeless tobacco had a longer primary delay and those with age less than 60 years had a longer secondary delay. The qualitative data identified the reasons for the diagnostic delay from the perspective of the patients. They were due to delay in symptom appraisal due to low perceived seriousness and delay in health-seeking. 

A tumor in the larynx could cause voice disturbance. This alteration in phonation could have adversely affected the livelihood and occupation of the patients prompting them to seek early health care. As the majority of the study population was within the working population (<60 years), this explanation could be the reason for the shorter primary delay in patients with laryngeal cancer when compared with oral or oropharyngeal cancer patients. Interestingly, laryngeal cancer patients had a longer secondary delay, though not statistically significant. This could be attributed to the fact that the histopathological diagnosis of such patients requires a biopsy from the lesion under general anesthesia. On comparison, a biopsy from the oral cavity and oropharynx were taken under local anesthesia in Minor operation theatre (OT) requiring less extensive pre-procedure investigations and obviating the need for major OT slot. Another interesting finding is that those patients who presented to JIPMER as their first health facility (without going anywhere else) had a higher primary delay and lower secondary dealy (Table 2), which is not statistically significant. This stand out finding can be explained with various hypothetical reasons.

The majority of the study participants were males (70.5%) and were in stage IV disease (60%), similar to the epidemiological profile of head and neck cancer in India (Kulkarni, 2013). The primary and secondary delay for head and neck cancer is lesser than that noted in other Indian studies done in the past. A study was done in New Delhi, India, in the year 2006-2007 found that median primary and secondary delay for a patient with head and neck cancer were 46 and 56 days, respectively (Dwivedi et al., 2012). In Maharashtra (2011-2012), the mean primary delay was 82 days (2.75 months), and the mean secondary delay was 58 days (1.94 months) (Joshi et al., 2014). Studies were done in other countries also noted that diagnostic delay was considerableand in most scenarios,the primary delay was higher than the secondary delay (Gigliotti et al., 2019; Stefanuto et al., 2014).However, in our study primary and secondary delays were similar. Our study participants were mostly from Tamil Nadu and Puducherry, a State/Union Territory with a good literacy rate and better health infrastructure. These factors along with improvement in health knowledge in recent years,could have attributed to shorter patient delay as compared to other studies. 

Age less than or equal to 60 years wasassociated with longer secondary delay. This may be due to a lower degree of suspicion of cancer by the health care staff among younger patients. Ever users of smokeless tobacco had a longer primary delay. It could be explained by qualitative results. Participants attributed their symptoms to addiction and did not feel a need to consult the health care professional for their symptoms. 

Diagnostic delays are due to both patient factors and system factors such as access, availability, and accessibility. Low socioeconomic status and other psychosocial factors such as patient beliefs on the cause of cancer and concerns on long term treatment influenced the duration of patient delays (Kumar et al., n.d.; Scott et al., 2006).The delay in symptom appraisal is mostly due to the low perceived seriousness of symptoms. Andersen et al. commented that the interpretation of bodily sensations as symptoms related to a specific cancer is embedded with cultural and social beliefs (Andersen et al., 2009).These were evident in the qualitative data. Patients reported the belief that symptoms are due to heat in the body caused by their occupation or due to addictions. We found that patients normalized and ignored the symptoms until it caused pain. In malignancy, pain appears at an advanced stage of cancer. Hence the other bodily sensations caused by the lesion are not perceived as serious by the participants. These beliefs lead to self-remedy and self-medication, thereby contributing to diagnostic delay. Similar results were observed by other qualitative research done to understand diagnostic delays (Azhar and Doss, 2018; Scott et al., 2006).

Noteworthy that symptom appraisal delay due to low perceived seriousness was mainly because of the low health literacy of the patients. A multi-centric community-based survey in India found that the awareness of cancer symptoms was low ranging from 4 to 22% for symptoms of head and neck cancer (Raj et al., 2012). In our study, participants regretted that they would have sort medical care early if they had known that the symptoms could be due to cancer. A similar low awareness of cancer symptoms is also noted in other countries (Al-Azri et al., 2015). In the interview, participants regretted and commented that they would have taken better care if they were aware of the possibility of cancer. Hence, improving health literacy will decrease the primary delay. 

In our study, delays in health-seeking were due to non-affordable health care costs, poor social support, self-remedy and self-medication. A qualitative study done in Malaysia found that self-remedy and self-medication were coping strategies that caused the delay in cancer diagnosis (Azharand Doss, 2018).

Reviews and meta-analysis have found that the association between diagnostic delay and tumor stage are inconclusive (Gigliotti et al., 2019; Gómez et al., 2009; Goy et al., 2009). But, a meta-analysis by Seoane et al., (2012) shows that diagnostic delay is a moderate risk factor for mortality in head and neck cancers. Measure taken to decrease diagnostic delay improves access to quality cancer care and to improve the overall survival rate of the patients.

The cancer control program should spread awareness of early warning symptoms.In addition, opportunistic screening should be encouraged. Adequate communication to the patient about the possibility of cancer by the doctor of first contact and strengthening of the referral system is essential. 

The diagnostic delay was considerable. Measures to enhance symptom appraisal by improving health literacy, opportunistic screening, and strengthening the referral system would decrease diagnostic delay. 
